# A consistent and potentially exploitable response during chondrogenesis of mesenchymal stem cells from osteoarthritis patients to the protein encoded by the susceptibility gene *GDF5*

**DOI:** 10.1371/journal.pone.0176523

**Published:** 2017-05-08

**Authors:** Madhushika Ratnayake, Maria Tselepi, Robert Bloxham, Frank Plöger, Louise N. Reynard, John Loughlin

**Affiliations:** 1 Musculoskeletal Research Group, Institute of Cellular Medicine, Newcastle University, Newcastle upon Tyne, United Kingdom; 2 BioPharm GmbH, Heidelberg, Germany; University of Alabama at Birmingham, UNITED STATES

## Abstract

Osteoarthritis (OA) is a common joint disease characterised by the focal loss of the protective cartilage layer at the ends of the bones. It is painful, disabling, multifactorial and polygenic. The growth differentiation factor 5 gene *GDF5* was one of the first reported OA susceptibility signals that showed consistent association to OA, with the transcript single nucleotide polymorphism (SNP) rs143383 demonstrating association in Asians and Europeans. The functional effect of the signal is reduced expression of the gene. The GDF5 protein is an extracellular matrix signalling molecule that is active during chondrogenesis and in mature chondrocytes. Due to the functional impact of the susceptibility, we previously assessed the effect of supplementing chondrocytes from OA patients with exogenous GDF5. Their response was highly discordant, precluding the application of GDF5 as a simple means of attenuating the genetic deficit. Since GDF5 is also active during development, we have now assessed the effect of exogenous GDF5 on bone marrow derived mesenchymal stem cells (MSCs) that are undergoing chondrogenesis during cartilage disc formation. MSCs from healthy donors and OA patients were studied and the effect of GDF5 was assessed by measuring the wet mass of the discs, by histological staining, and by monitoring the change in expression of anabolic, catabolic and hypertrophic protein-coding genes. The MSCs expressed the three principal GDF5 receptor genes and responded in a significantly anabolic manner (increase in wet mass, *p* = 0.0022; Bonferroni corrected *p* = 0.018) to a variant form of GDF5 that targets the most abundantly expressed receptor, BMPR-IA. GDF5 elicited significant (*p* < 0.05) changes in the expression of anabolic, catabolic and hypertrophic genes with several consistent effects in healthy donors and in OA patients. Our data implies that, unlike OA chondrocytes, OA MSCs do respond in a predictable, anabolic manner to GDF5, which could therefore provide a route to modulate the genetic deficit mediated by the rs143383 association signal.

## Introduction

Osteoarthritis (OA) is a painful and highly debilitating disease that principally affects older individuals. It is characterised by loss of articular cartilage that is accompanied by altered function of other synovial joint tissues. OA is multifactorial and polygenic, and is common in most human populations. One of the earliest reported loci to be reproducibly associated with OA susceptibility was the growth differentiation factor 5 gene *GDF5* [1]. Functional molecular and cellular investigations encompassing, amongst other experiments, luciferase reporter assays, allelic expression imbalance studies and DNA methylation assessments have implicated the single nucleotide polymorphism (SNP) rs143383 as the likely driver of this association signal [1–3]. rs143383 resides within the 5' untranslated region (5'UTR) of *GDF5*, with the risk-associated T allele of the SNP correlating with reduced expression of the gene [2].

GDF5 is a member of the bone morphogenetic protein (BMP) family and of the transforming growth factor β (TGFβ) superfamily. It has a primary role in skeletal formation, particularly during the early stages of chondrogenesis by regulating cell adhesion and chondrocyte proliferation in developing joints [4,5]. In humans, a heterozygous loss-of-function mutation in the gene results in a distinct subtype of brachydactyly, whereas a homozygous mutation is related to Grebe, Hunter-Thomspon and DuPan syndromes [6–8].

The members of the BMP family show different binding affinities for their receptors; GDF5 is known to bind with higher affinity to bone morphogenetic protein receptor type-1B (BMPR-IB) compared to bone morphogenetic protein receptor type-1A (BMPR-IA) and bone morphogenetic protein receptor type-2 (BMPR-II) [9]. A study by Zou and colleagues [10] suggests that BMPR-IB and BMPR-IA play distinct roles in different stages during limb development. BMPR-IB controls primary stages of mesenchyme condensation, preceding expression of the early cartilage marker genes *SOX9* and *COL2A1*, and promotes cartilage formation. In contrast, BMPR-IA regulates later chondrocyte differentiation, by acting downstream of indian hedgehog (IHH) in the prehypertrophic chondrocytes and by activating parathyroid hormone-related protein (PTHrP) expression [10].

Prompted by the functional impact that the rs143383 T allele association has on *GDF5* expression, we previously assessed the effect of GDF5 supplementation on primary chondrocytes that were extracted and grown from the cartilage of OA patients who had undergone hip or knee joint replacement surgery [11]. The inter-individual response however was highly discordant, precluding the application of exogenous GDF5 to chondrocytes as a simple means of attenuating the genetic deficit coded for by the *GDF5* association signal.

Human bone marrow derived mesenchymal stem cells (MSCs) have shown great potential to differentiate into multiple cell types, including the chondrogenic lineage; hence, they are considered strong candidates for tissue engineering and regeneration applications. Previous studies show that supplementation of GDF5 in MSC pellet cultures leads to an upregulation of chondrocyte specific genes such as *COL2A1* and *ACAN*, along with higher concentrations of extracellular matrix glycosaminoglycan (GAG), suggesting that GDF5 can promote chondrogenic differentiation of MSCs *in vitro* [12]. Murphy and colleagues [13] demonstrated that aggregate culture of MSCs from a healthy individual showed an upregulation of *COL2A1*, *ACAN* and *SOX9* in the presence of GDF5, TGFβ1 and BMP2. In addition, gene transfer of *GDF5* to MSCs by electroporation is reported to promote an intervertebral disc (IVD)-like phenotype and to increase *ACAN* and *SOX9* expression in a 3D organ culture model [14].

Murdoch and colleagues [15] have demonstrated that the Transwell culture system is a much more efficient model to induce chondrogenic differentiation of MSCs compared to other established protocols. The flat permeable arrangement of the Transwell insert supports a uniform nutrient supply to the MSCs, encouraging cell-cell contact, which is essential for chondrogenesis and which allows for cartilage disc formation. Their data report rapid cell proliferation, increased wet mass and strong extracellular matrix production over the first 7 days of differentiation, along with expression of cartilage genes, including *COL2A1*, *ACAN* and *SOX9*.

We have therefore explored the effect of exogenous GDF5 application on MSCs during the first 7 days of chondrogenic differentiation in the Transwell culture system. In addition to wildtype GDF5, we also assessed the effect of the GDF5 variant A form of the protein, which has increased specificity for BMPR-IA compared to BMPR-IB [11,16]. We studied MSCs isolated from healthy donors and from OA patients to determine if there were concordant responses within and between the two groups to GDF5 treatment during chondrogenesis. The cartilage discs were examined for differences in extracellular matrix production and for changes in the expression of genes that code for proteins involved in cartilage homeostasis. As far as we are aware, our analysis is also the largest study yet undertaken on the chondrogenic capacity of MSCs using the Transwell system, in that we investigated a total of 28 individuals (seven healthy donors and 21 OA patients). Our data also therefore provides pilot and exploratory attrition data on the efficacy of Transwell, in particular for OA MSCs.

## Materials and methods

### Ethics statement for obtaining hip joints from OA patients

The Newcastle and North Tyneside research ethics committee granted ethical approval for the collection of hip joints from patients undergoing total hip replacement for primary OA (REC reference number 14/NE/1212). The ethics committee approved the consent procedure, in which a trained research nurse discussed the project with the patient, and if the patient agreed to participate, a written informed consent was taken and filed by the consenting nurse. OA status was confirmed using pre-operative clinical records and all patients had full-thickness cartilage lesions.

### Mesenchymal stem cell (MSC) isolation and culture

Bone marrow derived MSCs isolated from the iliac crest of healthy donors were purchased from Lonza and were expanded in mesenchymal stem cell basal media (MSCBM; Lonza) containing 5 ng/ml human FGF2 (R&D Systems).

Bone marrow derived MSCs were extracted from the femoral head of OA patients following the protocol outlined by Neagu and colleagues [17]. Bone fragments from the marrow cavity of the femoral head were extracted into 10 ml of phosphate-buffered saline (PBS; Sigma) and layered on to 10 ml of Ficoll-Plaque Premium (GE Healthcare) and centrifuged at 800 x *g* for 40 minutes. The mononuclear cell layer was extracted from the cell suspension and washed in PBS containing 0.2% bovine serum albumin (BSA; Sigma) and 5 mM EDTA (Sigma) and centrifuged at 240 x *g* for 10 minutes. The cell pellet was resuspended in DMEM (Sigma) containing 10% FBS (Sigma), 2 mM GlutaMAX (Life Technologies), 100 U/ml penicillin and 100 μg/ml streptomycin (Sigma) and seeded in a T-25 cell culture flask. The medium was replaced after 24 hours and then every 3 days until the cells reached 80% confluence. The cells were passaged and cultured in MSCBM supplemented with 5 ng/ml FGF2.

### MSC chondrogenic differentiation and exposure to exogenous GDF5

Healthy donor and patient MSCs were cultured in MSCBM containing 5 ng/ml FGF2 until 80% confluent. The cells were trypsinised, centrifuged at 240 x *g* for 5 minutes and were differentiated into chondrocytes using the Transwell protocol [15]. For each cartilage disc, 500,000 cells were used and the cells were resuspended in differentiation media with or without 100 ng/ml wildtype GDF5 or GDF5 variant A; we used 100 ng/ml of GDF5 as our previous experiments had demonstrated a robust response of chondrocytes to this concentration of the growth factor [11]. The wildtype and variant A forms of GDF5 protein were provided by BioPharm GmbH, Heidelberg, Germany. The differentiation media consisted of DMEM containing 4.5 mg/ml glucose (Lonza, catalogue number 12–614), 10 ng/ml TGF-β3 (PeproTech), 100 mM dexamethasone (Sigma), 50 μg/ml ascorbic acid-2-phosphate (Sigma), 40 μg/ml proline (USB), 1 x Insulin, Transferin, Selenium, Linoleic acid premix (ITS+L; BD Biosciences), 20 μM glutamine (Sigma), 1 U/ml penicillin and 100 μg/ml streptomycin mix (Sigma). Millicell 0.4 μm hanging PET cell culture inserts (Merck Millipore, catalogue number PIHT12L04) were placed in a 24-well plate and 500,000 MSCs in 300 μl of media were transferred to each insert. The plates were centrifuged at 200 x *g* for 5 minutes and 650 μl of differentiation media were added to the bottom of the wells. For each donor the discs were to be harvested at two different time points (days 3 and 7), therefore six Transwells were used in total; untreated, treated with wildtype GDF5 and treated with variant A. At each time point, the discs were harvested and the whole disc was weighed on parafilm to obtain the wet mass. The discs were stored at -80°C until all time points were reached.

### Histology

Due to their larger size, histology was performed only on the day 7 cartilage discs. One half of a disc was sectioned, fixed in 10% formalin solution (CellPath) overnight and wax embedded following standard processing protocols. Four μm transverse sections were taken, with one section from each treatment mounted onto each Cellstik Superfrost Ultra Plus slide (CellPath). Slides were stained with haematoxylin and eosin (Licor), Safranin-O (Sigma) and Masson’s Trichrome (Sigma) and images were taken using a Leica DM4000B light microscope at 20 x magnification.

### RNA extraction from cartilage discs

All of each day 3 disc and the remaining half of each day 7 disc were used for RNA extraction. The discs were ground using a pestle in 250 μl of Trizol reagent (Ambion). RNA was isolated using the Trizol/chloroform method according to manufacturer’s guidelines (Invitrogen). Complementary DNA (cDNA) was then synthesised from 500 ng of RNA using the SuperScript First-Strand Synthesis System (Invitrogen) and the manufacturer’s guidelines.

### Gene expression analysis using quantitative real time PCR

The cDNA was diluted 1 in 20 with distilled water (Sigma). TaqMan primers and probes were used to analyse gene expression changes in a panel of anabolic, catabolic and hypertrophic genes (S1 Table). Gene expression was measured in triplicate and normalised to the average expression of the housekeeping genes *18S*, *GAPDH* and *HPRT1*. Reactions were performed on an ABI PRISM 7900HT Real Time PCR System. The relative expression for each gene was analysed using the comparative cycle threshold (Ct) method using SDS 2.3 software (Applied Biosystems). Gene expression relative to the housekeeping genes was calculated using the formula 2^-(Ct of target gene–average Ct of the three housekeeping genes). The fold change in gene expression between the untreated and GDF5 treated cells was calculated for each sample at all time points. A Wilcoxon signed rank test was then performed on the fold change in gene expression for each gene analysed, to determine if the data deviated significantly from a value of 1.0, representing untreated (control). *P* values less than 0.05 were considered significant.

## Results

### Assessing the capacity of MSCs to expand and then undergo chondrogenic differentiation

Over a period of two years we purchased MSCs from seven healthy donors and extracted MSCs from 21 OA patients (Table 1). Each MSC was then subjected to culture and expansion. This was successful for all of the healthy donors and for 11 of the OA patients. There were no clear differences between the successful and unsuccessful OA patients with regard to age or sex. The expanded MSCs were then subjected to chondrogenic differentiation into cartilage discs. This was successful for three of the seven healthy donors and for four of the 11 OA patients. These seven individuals, given the laboratory identification (ID) letters A-G, were then taken forward for subsequent analysis.

**Table 1:** The 28 donors and patients initially studied.

Donor/ Patient	ID used in this report	Phenotype	Sex	Age	MSC origin	MSC expansion	Chondrogenic differentiation
1	A	Healthy	F	24	IC	Yes	Yes
2	B	Healthy	F	41	IC	Yes	Yes
3	C	Healthy	M	25	IC	Yes	Yes
4	-	Healthy	M	29	IC	Yes	No
5	-	Healthy	F	18	IC	Yes	No
6	-	Healthy	M	38	IC	Yes	No
7	-	Healthy	F	29	IC	Yes	No
8	D	OA	F	69	FH	Yes	Yes
9	E	OA	F	81	FH	Yes	Yes
10	F	OA	M	55	FH	Yes	Yes
11	G	OA	F	67	FH	Yes	Yes
12	-	OA	F	44	FH	Yes	No
13	-	OA	M	41	FH	Yes	No
14	-	OA	F	81	FH	Yes	No
15	-	OA	M	92	FH	Yes	No
16	-	OA	F	73	FH	Yes	No
17	-	OA	F	51	FH	Yes	No
18	-	OA	M	64	FH	Yes	No
19	-	OA	F	81	FH	No	n/a
20	-	OA	F	61	FH	No	n/a
21	-	OA	F	80	FH	No	n/a
22	-	OA	M	69	FH	No	n/a
23	-	OA	F	65	FH	No	n/a
24	-	OA	F	68	FH	No	n/a
25	-	OA	F	71	FH	No	n/a
26	-	OA	M	71	FH	No	n/a
27	-	OA	F	56	FH	No	n/a
28	-	OA	F	75	FH	No	n/a

F, female. M, male. Age (in years) is the age of the donor or patient when the MSCs were harvested. IC, iliac crest. FH, femoral head. Yes, MSC expansion or chondrogenic differentiation was successful. No, MSC expansion or chondrogenic differentiation was unsuccessful. n/a, not applicable, chondrogenic differentiation could not be undertaken if MSC expansion failed.

### GDF5 receptor genes are expressed in cartilage discs grown from healthy donor and OA MSCs

**Fig 1:**
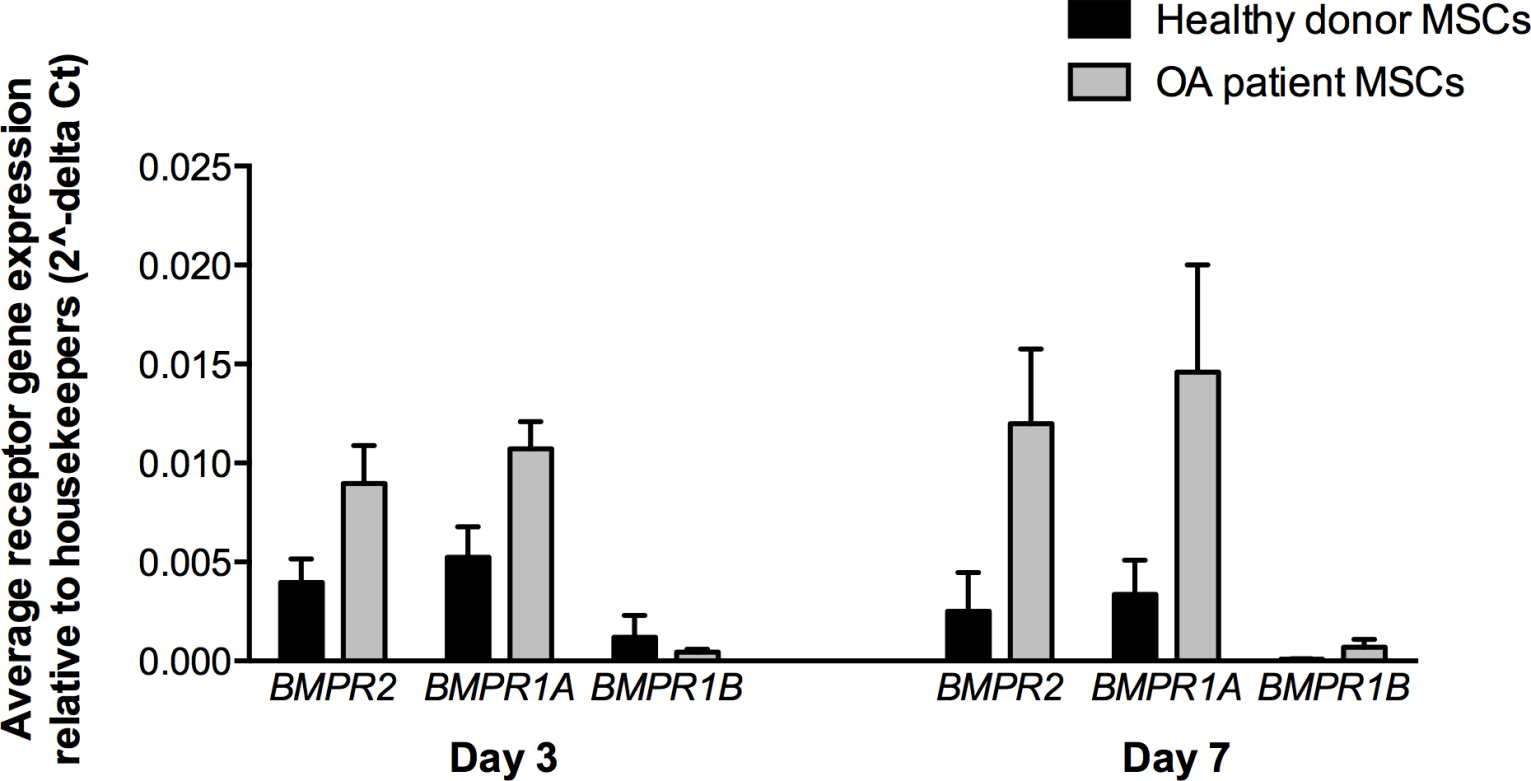
Expression of the GDF5 receptor genes. Expression was measured in cartilage discs grown from healthy donor MSCs (black bars; n = 3) and from OA patient MSCs (grey bars; n = 4) at days 3 and 7. Discs were grown without exposure to exogenous GDF5. Expression was assessed relative to the housekeeping genes *18S*, *GAPDH* and *HPRT1*. Three technical repeats were performed for each gene per donor/patient. The error bars represent the standard error of the mean.

Using quantitative real time PCR, we measured the expression of *BMPR2*, *BMPR1A* and *BMPR1B*, which encode GDF5 receptors BMPR-II, BMPR-IA and BMPR-IB, respectively. Our previous studies in primary chondrocytes from OA patients showed that expression of *BMPR1B*, which codes for the BMPR-IB receptor that GDF5 preferentially binds to, was up to 65 fold lower than the expression of *BMPR1A* [11]. The MSC-derived cartilage discs from the healthy donors and from the OA patients expressed all three receptor genes, with higher levels of *BMPR2* and *BMPR1A* relative to *BMPR1B* (Fig 1 and S1 Fig). *BMPR1A* expression was on average 676 and 24 fold higher than *BMPR1B* in discs derived from healthy donor MSCs at day 3 and 7 respectively, and 50 and 21 fold higher in discs derived from OA patient MSCs at day 3 and 7 respectively. These results therefore match those observed for primary chondrocytes and justified our decision to study both the wildtype form of GDF5 and the variant A form, since this latter version of the protein binds preferentially to the BMPR-IA receptor [16]. This version of the GDF5 protein is henceforth referred to as GDF5 variant.

### Effect of exogenous GDF5 on cartilage extracellular matrix as assessed by wet mass and histology of the cartilage discs

As MSCs differentiate towards chondrocytes a highly hydrated extracellular matrix is deposited in the discs [18]. This is an anabolic response and reflects an increased GAG content of the matrix; in normal physiological circumstances, the GAG binds water leading to the swelling observed in the healthy cartilage that enables the tissue to resist load. As a proxy for a positive anabolic response, we therefore measured the wet mass of the untreated (control) discs and of the discs exposed to wildtype GDF5 and to GDF5 variant. The experiment was performed on two of the three healthy donor MSCs (donors B and C) and on all four of the OA patient MSCs (patients D-G) at days 3 and 7 of chondrogenesis.

**Fig 2:**
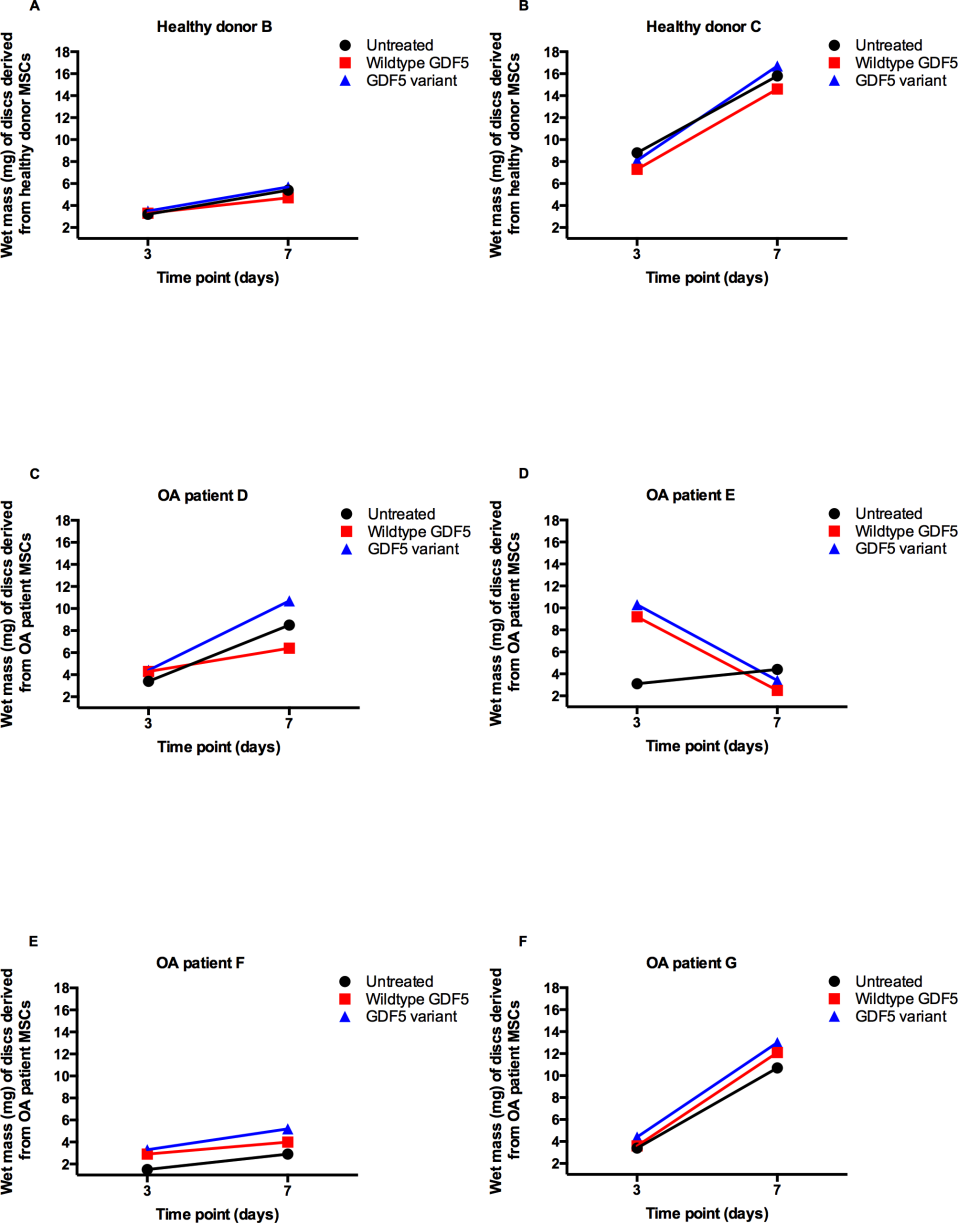
The change in wet mass of the cartilage discs in response to GDF5. The wet mass (mg) of discs grown from healthy donor MSCs (graphs A and B) and from OA patient MSCs (graphs C-F) was measured at days 3 and 7. Discs were grown without exogenous GDF5 (untreated, black lines), with wildtype GDF5 (red lines), or with GDF5 variant (blue lines).

The wet mass of the discs from the two healthy donors and from three of the four OA patients (D, F and G) increased during the differentiation time course with or without exogenous GDF5 (Table 2 and Fig 2A–2C, 2E and 2F). The exception was patient E (Fig 2D), in whom the exogenous GDF5 treatment resulted in a striking increase in disc mass at day 3 relative to untreated, but by day 7 the treated discs had lost mass and were lighter than the untreated disc.

**Table 2:** The wet mass of the cartilage discs differentiated from MSCs derived from healthy donors (B and C) and OA patients (D-G).

		Wet mass of discs (mg) on day 3			Wet mass of discs (mg) on day 7		
Donor/Patient	Phenotype	Untreated	Wildtype GDF5	Variant form GDF5	Untreated	Wildtype GDF5	Variant form GDF5
B	Healthy	3.2	3.3	3.5	5.4	4.7	5.7
C	Healthy	8.8	7.3	8.1	15.8	14.6	16.7
D	OA	3.4	4.3	4.4	8.5	6.4	10.7
E	OA	3.1	9.2	10.3	4.4	2.5	3.4
F	OA	1.5	2.9	3.3	2.9	4.0	5.2
G	OA	3.4	3.6	4.4	10.7	12.1	13.0

Wet mass (mg) was measured at day 3 and 7 of chondrogenesis. Discs were grown without exogenous GDF5 (untreated) and with wildtype (wt) or variant form of exogenous GDF5.

The wet mass of donor B (Fig 2A) was less than donor C (Fig 2B) for both time points, suggesting inter-individual differences in the chondrogenic potential between the two healthy MSCs. Similar inter-individual differences were also observed for the OA patients.

Interestingly, for all of the two donors and four patients the wet mass at both day 3 and day 7 was greater when the cells were exposed to GDF5 variant than when they were exposed to wildtype GDF5. The probability of observing this by chance alone is small for the combined data of day 3 and day 7 (*p* = 0.0022; one-tailed paired t-test), and when each day is assessed separately (*p* = 0.0084 for day 3 and 0.012 for day 7; one-tailed paired t-test). In a comparison of untreated versus treatment with GDF5 variant, for five of the six donors the wet mass was greater following exposure to GDF5 variant at day 3 (donor B and patients D-G) and at day 7 (donor B and C, patients D, F and G). These results were significant for the combined data for day 3 and 7 (*p* = 0.017; one-tailed paired t-test), for day 7 alone (*p* = 0.044; one-tailed paired t-test) and approached significance for day 3 (*p* = 0.091; one-tailed paired t-test). There were no significant differences for untreated versus wildtype GDF5 (all *p* values >0.05). As noted in the previous section, *BMPR1A*, which codes for the receptor that GDF5 variant preferentially binds to, is more highly expressed in the cartilage discs than are the other GDF5 receptor genes (Fig 1). This data implies therefore that the discs respond in a consistently anabolic manner to GDF5 variant and that this may be a reflection of the increased expression by the chondrocytes of the appropriate receptor for this form of the growth factor.

**Fig 3:**
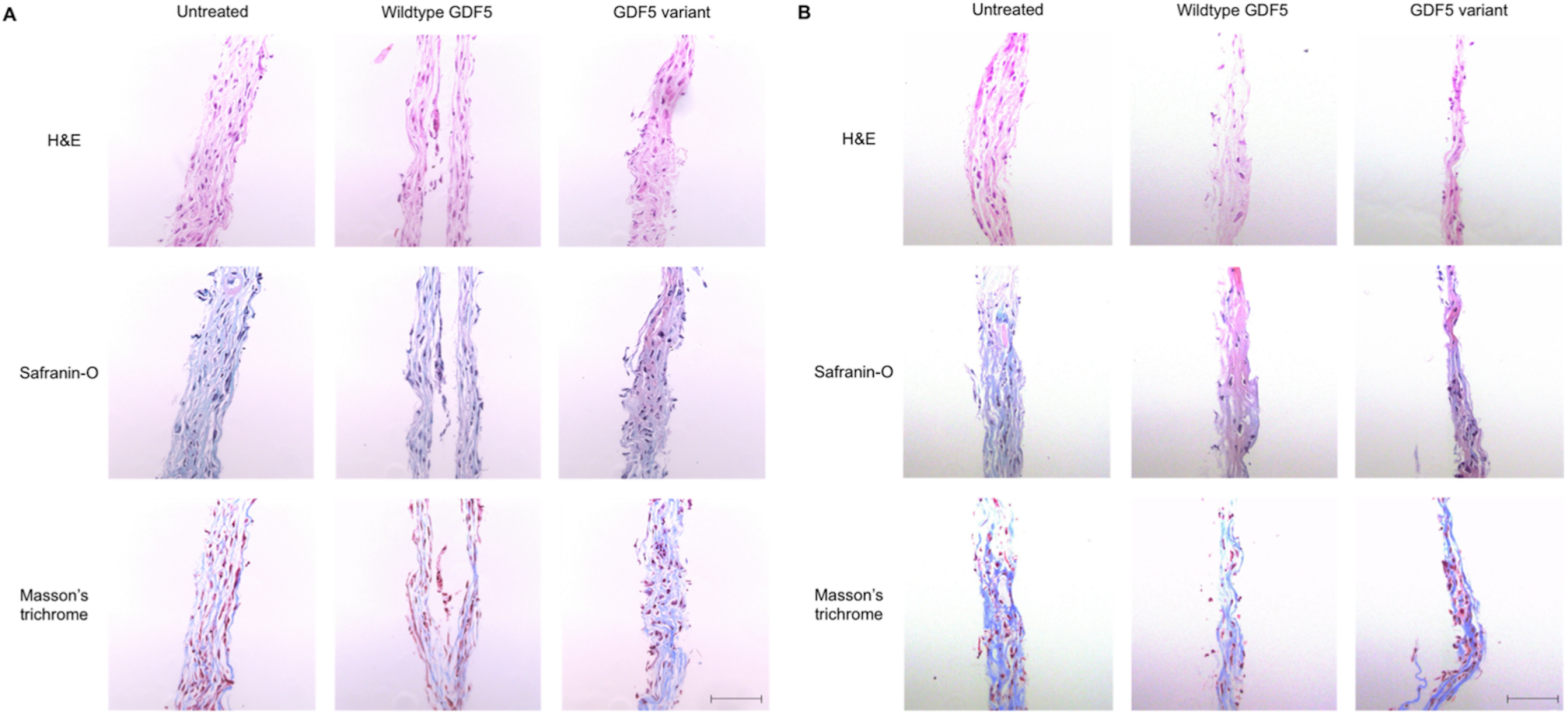
Histology of cartilage discs at day 7 of chondrogenesis. Discs grown from the MSCs of a healthy donor (donor C; panel A) and an OA patient (patient D; panel B) are shown and are representative of the separate experiments carried out for each donor/patient. Four μm sections were taken and stained with haemotoxylin and eosin (H&E; top row), Safranin-O (middle row) and Masson’s Trichrome (bottom row). The black horizontal bar represents a 100 μm scale. Images were taken at 20 x magnification.

We next performed histology on the day 7 discs using haemotoxylin and eosin (H&E) to stain for cells, Safranin-O to stain for cartilage proteoglycan and Masson’s trichrome to stain for collagen. We observed positive staining for all three stains in all of our samples, suggesting that the discs produced an extracellular matrix rich in proteoglycan and collagen. We did not take any objective measures and we observed no subjective differences according to the origin of the MSCs (healthy or OA), or to treatment of the discs (untreated versus treated with wildtype GDF5 or GDF5 variant). Fig 3A and 3B provide representative results from healthy donor C and OA patient D, respectively.

### Effect of exogenous GDF5 on gene expression during chondrogenesis of MSCs from the healthy donors

Tables 3 and 4 list the average fold change in gene expression of each of the target genes in each of the three healthy donors in response to wildtype GDF5 and GDF5 variant, respectively. The fold changes are relative to untreated (control) and the data is presented for both day 3 and day 7 discs. The data for all three donors was then combined to assess if there were any consistent effects across the donors; Table 5 lists the *p* values obtained with the Wilcoxon signed rank test.

**Table 3:** The fold change in expression of anabolic, catabolic and hypertrophic genes in the cartilage discs from each healthy donor in response to wildtype GDF5.

Fold change in gene expression in response to wildtype GDF5 in healthy donors A-C							
Role	Gene	Day 3			Day 7		
		Donor A	Donor B	Donor C	Donor A	Donor B	Donor C
Anabolic	*ACAN*	1.99	1.59	1.09	1.69	0.98	0.83
	*COL2A1*	2.58	1.08	0.60	2.03	1.32	0.63
	*SOX9*	0.50	1.42	1.10	1.01	1.08	1.40
	*TIMP1*	0.63	1.40	1.21	0.68	1.21	1.38
Catabolic	*ADAMTS4*	1.02	1.24	1.20	0.17	0.83	3.81
	*ADAMTS5*	0.58	0.96	1.68	0.28	1.29	2.98
	*MMP1*	0.86	1.40	1.31	1.70	1.09	0.95
	*MMP13*	1.12	0.84	0.77	1.06	2.82	1.70
Hypertrophic	*BGLAP*	0.68	0.84	1.45	1.00	1.13	0.68
	*COL1A1*	0.82	0.67	0.84	0.57	1.26	0.68
	*COL10A1*	0.89	1.03	0.60	1.71	1.06	0.20
	*IHH*	0.37	1.10	1.05	1.28	1.35	0.18
	*RUNX2*	0.75	1.21	0.99	1.45	0.90	0.71
	*RUNX2* bn	0.63	1.19	1.02	0.82	1.41	0.59

Gene expression was measured at day 3 and day 7 of chondrogenesis without exogenous GDF5 (untreated) or with wildtype GDF5. A fold change of 1 would represent no change in target gene expression relative to untreated. A value greater than 1 (highlighted in green) denotes increased gene expression relative to untreated and a value less than 1 (highlighted in red) denotes decreased gene expression relative to untreated. *RUNX2* bn, the bone specific *RUNX2* transcript.

**Table 4:** The fold change in expression of anabolic, catabolic and hypertrophic genes in the cartilage discs from each healthy donor in response to variant A form of GDF5.

Fold change in gene expression in response to variant A form of GDF5 in healthy donors A-C							
Role	Gene	Day 3			Day 7		
		Donor A	Donor B	Donor C	Donor A	Donor B	Donor C
Anabolic	*ACAN*	0.98	0.82	0.79	0.62	1.48	0.63
	*COL2A1*	3.24	1.10	0.73	0.80	1.52	0.50
	*SOX9*	1.06	1.05	0.78	1.23	1.58	1.59
	*TIMP1*	0.87	1.04	1.13	0.87	1.37	1.16
Catabolic	*ADAMTS4*	0.83	0.86	1.05	1.26	1.00	6.64
	*ADAMTS5*	1.02	0.99	1.54	1.09	1.28	2.98
	*MMP1*	0.41	1.17	1.67	0.76	2.12	0.95
	*MMP13*	0.45	0.72	0.65	0.74	2.87	2.99
Hypertrophic	*BGLAP*	1.02	0.56	0.65	1.58	0.58	0.47
	*COL1A1*	1.85	1.26	0.77	1.22	2.12	0.52
	*COL10A1*	3.90	1.33	0.75	0.94	1.06	0.18
	*IHH*	6.71	1.64	0.71	0.45	1.79	0.11
	*RUNX2*	1.31	1.05	0.88	1.69	1.03	0.71
	*RUNX2* bn	1.39	1.09	1.09	1.69	1.10	0.76

Gene expression was measured at day 3 and day 7 of chondrogenesis without exogenous GDF5 (untreated) or with variant A form of GDF5. A fold change of 1 would represent no change in target gene expression relative to untreated. A value greater than 1 (highlighted in green) denotes increased gene expression relative to untreated and a value less than 1 (highlighted in red) denotes decreased gene expression relative to untreated. *RUNX2* bn, the bone specific *RUNX2* transcript.

**Table 5:** The *p* values calculated using the Wilcoxon signed rank test for each gene in the three healthy donor cartilage discs in response to GDF5.

A	Wildtype GDF5		B	GDF5 variant	
Gene	Time point		Gene	Time point	
	Day 3	Day 7		Day 3	Day 7
*ACAN*	**0.0078 (inc)**	0.50	*ACAN*	**0.023 (dec)**	0.82
*COL2A1*	0.50	0.25	*COL2A1*	0.50	0.91
*SOX9*	0.69	**0.027 (inc)**	*SOX9*	1.00	**0.0039 (inc)**
*TIMP1*	0.81	0.36	*TIMP1*	0.94	0.16
*ADAMTS4*	**0.016 (inc)**	0.91	*ADAMTS4*	0.13	**0.019 (inc)**
*ADAMTS5*	0.65	0.30	*ADAMTS5*	0.22	**0.039 (inc)**
*MMP1*	0.22	0.43	*MMP1*	0.94	0.74
*MMP13*	0.31	**0.016 (inc)**	*MMP13*	**0.0078 (dec)**	0.055
*BGLAP*	0.81	0.94	*BGLAP*	0.58	1.00
*COL1A1*	**0.012 (dec)**	0.38	*COL1A1*	0.078	0.11
*COL10A1*	0.13	1.00	*COL10A1*	0.098	0.25
*IHH*	0.43	0.64	*IHH*	0.15	0.84
*RUNX2*	0.58	1.00	*RUNX2*	0.38	0.57
*RUNX2* bn	0.69	1.00	*RUNX2* bn	**0.031 (inc)**	0.30

Data derives from Tables 3 and 4. Comparisons were between untreated (control) and treatment with either exogenous wildtype GDF5 (A) or with GDF5 variant (B). *P* values <0.05 and arising from fold changes in the same direction for all three donors are highlighted in bold. *RUNX2* bn, bone specific *RUNX2* transcript. (inc), increased expression; (dec), decreased expression.

It is clear from Tables 3 and 4 that there are a range of responses to both forms of GDF5, with the largest fold increase being 6.64 for *ADAMTS4* at day 7 in donor C and in response to GDF5 variant, and the largest fold decrease being 0.11 (9.09 fold) for *IHH*, also at day 7, also in donor C and also in response to GDF5 variant.

Many of the gene expression changes are in the same direction for all three donors and several of these are significant. For wildtype GDF5 (Table 5A), a significant increase in expression was observed across the three donors for *ACAN* (*p* = 0.0078, day 3), *ADAMTS4* (*p* = 0.016, day 3), *SOX9* (*p* = 0.027, day 7) and *MMP13* (*p* = 0.016, day 7), and a significant decrease was observed for *COL1A1* (*p* = 0.012, day 3). For GDF5 variant (Table 5B), a significant increase in expression was observed across the three donors for the *RUNX2* bone specific transcript *RUNX2* bn (*p* = 0.031, day 3), for *SOX9* (*p* = 0.0039, day 7), *ADAMTS4* (*p* = 0.019, day 7) and *ADAMTS5* (*p* = 0.039, day 7), and a significant decrease was observed for *ACAN* (*p* = 0.023, day 3) and *MMP13* (*p* = 0.0078, day 3).

*SOX9*, which encodes the key anabolic chondrogenic transcription factor, was the only gene that showed a consistent, significant change in expression across the three donors, at the same time point and in response to both forms of GDF5, with the gene showing an increased expression at day 7 (*p* = 0.027 for wildtype GDF5 and 0.0039 for GDF5 variant; Table 5). The average fold increase for *SOX9* was greater in response to GDF5 variant than in response to wildtype GDF5; the individual donor increases for variant were 1.23, 1.58 and 1.59 (Table 4), making an average of 1.47, whereas the individual donor increases for wildtype were 1.01, 1.08 and 1.40 (Table 3), making an average of 1.16.

### Effect of exogenous GDF5 on gene expression during chondrogenesis of MSCs from the OA patients

Tables 6 and 7 list the average fold change in gene expression of each of the target genes in each of the four OA patients in response to wildtype GDF5 and GDF5 variant, respectively. As above, the fold changes are relative to untreated (control) and the data is presented for day 3 and day 7 discs. The data was then combined to assess if there were any consistent effects across all four patients; Table 8 lists the *p* values obtained with the Wilcoxon signed rank test.

**Table 6:** The fold change in expression of anabolic, catabolic and hypertrophic genes in the cartilage discs from each OA patient in response to wildtype GDF5.

Fold change in gene expression in response to wildtype GDF5 in OA patients D-G									
Role	Gene	Day 3				Day 7			
		Patient D	Patient E	Patient F	Patient G	Patient D	Patient E	Patient F	Patient G
Anabolic	*ACAN*	1.11	0.26	1.72	2.31	1.78	0.52	1.98	0.53
	*COL2A1*	1.22	0.73	3.84	2.80	2.34	0.31	2.16	0.39
	*SOX9*	1.07	0.42	1.20	1.51	1.39	0.60	1.57	0.90
	*TIMP1*	1.07	1.22	0.79	1.36	1.03	1.48	0.97	0.96
Catabolic	*ADAMTS4*	0.94	0.77	0.86	1.65	0.83	0.98	0.71	0.79
	*ADAMTS5*	0.53	2.81	0.53	1.62	0.62	1.14	2.73	0.79
	*MMP1*	2.41	0.80	0.90	1.49	0.90	0.23	0.19	0.37
	*MMP13*	1.89	1.05	1.09	2.48	0.89	0.81	0.73	0.73
Hypertrophic	*BGLAP*	0.82	0.88	0.74	1.17	0.95	1.02	1.09	1.34
	*COL1A1*	0.83	0.65	1.01	1.24	1.04	0.81	2.64	0.99
	*COL10A1*	0.83	1.43	1.95	2.32	1.82	0.65	2.16	1.05
	*IHH*	0.80	0.41	2.51	2.95	2.05	0.31	0.87	0.92
	*RUNX2*	0.86	1.78	0.81	1.10	0.91	0.76	1.20	0.86
	*RUNX2* bn	1.32	1.89	1.01	1.37	0.83	1.01	1.35	0.57

Gene expression was measured at day 3 and day 7 of chondrogenesis without exogenous GDF5 (untreated) or with wildtype GDF5. A fold change of 1 would represent no change in target gene expression relative to untreated. A value greater than 1 (highlighted in green) denotes increased gene expression relative to untreated and a value less than 1 (highlighted in red) denotes decreased gene expression relative to untreated. *RUNX2* bn, the bone specific *RUNX2* transcript.

**Table 7:** The fold change in expression of anabolic, catabolic and hypertrophic genes in the cartilage discs from each OA patient in response to variant A form of GDF5.

Fold change in gene expression in response to variant A form of GDF5 in OA patients D-G									
Role	Gene	Day 3				Day 7			
		Patient D	Patient E	Patient F	Patient G	Patient D	Patient E	Patient F	Patient G
Anabolic	*ACAN*	1.41	0.18	2.89	3.80	2.95	0.54	0.34	0.62
	*COL2A1*	2.53	0.26	7.31	8.14	2.13	0.37	0.32	0.50
	*SOX9*	1.22	0.25	1.21	2.60	2.19	0.52	1.02	1.08
	*TIMP1*	1.24	0.89	0.89	2.00	0.39	2.39	1.14	0.31
Catabolic	*ADAMTS4*	0.98	0.63	0.83	1.62	0.85	1.10	1.17	0.43
	*ADAMTS5*	0.51	3.23	0.29	2.25	0.25	2.67	5.26	0.64
	*MMP1*	2.24	0.76	1.43	2.01	1.65	4.18	0.27	0.19
	*MMP13*	3.81	1.76	1.45	3.38	0.57	1.87	5.23	0.35
Hypertrophic	*BGLAP*	0.59	0.87	1.47	2.13	0.60	2.15	1.62	1.36
	*COL1A1*	0.75	0.54	0.54	1.55	1.84	0.84	0.87	0.94
	*COL10A1*	0.69	1.69	1.20	4.77	1.03	1.46	0.61	0.62
	*IHH*	0.62	0.29	1.64	7.47	3.02	0.37	0.09	0.36
	*RUNX2*	1.04	1.43	1.06	1.31	0.58	1.31	1.89	0.90
	*RUNX2* bn	1.23	1.78	1.19	1.08	0.57	2.22	1.98	0.64

Gene expression was measured at day 3 and day 7 of chondrogenesis without exogenous GDF5 (untreated) or with variant A form of GDF5. A fold change of 1 would represent no change in target gene expression relative to untreated. A value greater than 1 (highlighted in green) denotes increased gene expression relative to untreated and a value less than 1 (highlighted in red) denotes decreased gene expression relative to untreated. *RUNX2* bn, the bone specific *RUNX2* transcript.

**Table 8:** The *p* values calculated using the Wilcoxon signed rank test for each gene in the four OA patient cartilage discs in response to GDF5.

A	Wildtype GDF5		B	GDF5 variant	
Gene	Time point		Gene	Time point	
	Day 3	Day 7		Day 3	Day 7
*ACAN*	0.11	0.18	*ACAN*	0.064	0.68
*COL2A1*	*0*.*034 (inc)*	0.32	*COL2A1*	*0*.*0068 (inc)*	0.68
*SOX9*	0.52	0.38	*SOX9*	0.32	0.47
*TIMP1*	0.15	0.38	*TIMP1*	0.23	0.97
*ADAMTS4*	0.79	**0.0049 (dec)**	*ADAMTS4*	0.79	0.42
*ADAMTS5*	0.24	0.79	*ADAMTS5*	0.34	0.32
*MMP1*	0.27	**0.034 (dec)**	*MMP1*	*0*.*037 (inc)*	0.73
*MMP13*	**0.0068 (inc)**	**0.0034 (dec)**	*MMP13*	**0.0005 (inc)**	0.68
*BGLAP*	0.62	0.15	*BGLAP*	0.74	0.23
*COL1A1*	0.23	0.52	*COL1A1*	0.57	0.85
*COL10A1*	*0*.*012 (inc)*	0.064	*COL10A1*	*0*.*027 (inc)*	0.42
*IHH*	0.18	0.73	*IHH*	0.52	0.68
*RUNX2*	0.52	0.083	*RUNX2*	**0.0098 (inc)**	0.70
*RUNX2* bn	**0.0093 (inc)**	0.58	*RUNX2* bn	**0.0098 (inc)**	0.12

Data derives from Tables 6 and 7. Comparisons were between untreated (control) and treatment with either exogenous wildtype GDF5 (A) or with GDF5 variant (B). *P* values <0.05 and arising from fold changes in the same direction for all four patients are highlighted in bold. *P* values <0.05 but arising from fold changes in the same direction for only three of the four patients are highlighted in italic. *RUNX2* bn, bone specific *RUNX2* transcript. (inc), increased expression; (dec), decreased expression.

As for the healthy MSCs, there are a range of responses to both forms of GDF5 by the OA MSCs, with the largest fold increase being 7.47 for *IHH* at day 3 in patient G and in response to GDF5 variant, and the largest fold decrease being 0.09 (11.11 fold) for *IHH* at day 3 in patient F and in response to GDF5 variant (Table 7). It is potentially noteworthy that for both the healthy MSCs and the OA MSCs, *IHH* showed the largest fold decrease in expression in any one donor or patient, and that this change was in response to GDF5 variant.

Many of the gene expression changes observed are in the same direction for all four OA patients and several of these were significant. For wildtype GDF5 (Table 8A), a significant increase in expression was observed across the four patients for *MMP13* (*p* = 0.0068, day 3) and *RUNX2* bn (*p* = 0.0093, day 3), and a significant decrease was observed for *ADAMTS4* (*p* = 0.0049, day 7), *MMP1* (*p* = 0.034, day 7) and *MMP13* (*p* = 0.0034, day 7). For GDF5 variant (Table 8B), a significant increase in expression was observed across the four patients for *MMP13* (*p* = 0.0005, day 3), *RUNX2* (*p* = 0.0098, day 3) and *RUNX2* bn (*p* = 0.0098, day 3).

At day 3 a significant trend of increased expression was observed for *COL2A1* (*p* = 0.034) and *COL10A1* (*p* = 0.012) with wildtype GDF5, and for *COL2A1* (*p* = 0.0068), *MMP1* (*p* = 0.037) and *COL10A1* (*p* = 0.027) with GDF5 variant. However, these significant effects (which are highlighted in italic in Table 8) were only consistent amongst three of the four OA patients (Tables 6 and 7), and not the same three patients each time, implying that there was not a patient behaving aberrantly relative to the others in these particular comparisons.

In the healthy donors, *SOX9* demonstrated a consistent, significant change in expression across the donors, at the same time point and in response to both forms of GDF5 (Tables 3, 4 and 5). That was not the case in the OA patients. Two genes did however demonstrate such a consistent expression change across all OA patients at the same time point and in response to both forms of GDF5; *MMP13* and *RUNX2* bn, which both demonstrated a significant increase in expression at day 3 (for *MMP13*, *p* = 0.0068 for wildtype GDF5 and 0.0005 for GDF5 variant; for *RUNX2* bn, *p* = 0.0093 for wildtype GDF5 and 0.0098 for GDF5 variant; Table 8).

If we compare healthy MSCs to OA MSCS, the gene that showed a consistent, significant change in expression at the same time point and in response to exogenous GDF5 was the bone specific *RUNX2* bn transcript, the expression of which was increased significantly at day 3 in response to GDF5 variant (for healthy MSCs, *p* = 0.031 and for OA MSCs, *p* = 0.0098; Tables 5 and 8). The gene that showed the greatest number of significant responses across all donors/patients was *MMP13*, which demonstrated a significant change in five of the eight comparisons made; two of four comparisons for the healthy MSCs (Table 5) and three of four comparisons for the OA MSCs (Table 8). However, the response to the two forms of GDF5 was not in the same direction in all of the comparisons. For healthy MSCs, *MMP13* expression was increased at day 7 in response to wildtype GDF5, whereas there was reduced expression at day 3 in response to GDF5 variant (Table 5). Similarly, for the OA MSCs, *MMP13* expression was increased at day 3 in response to both wildtype and GDF5 variant, and was decreased at day 7 in response to wildtype GDF5 (Table 8).

There were nine genes in total that showed a significant change either in the healthy or the OA MSCs (*ACAN*, *SOX9*, *ADAMTS4*, *ADAMTS5*, *MMP1*, *MMP13*, *COL1A1*, *RUNX2* and *RUNX2* bn). Of these nine genes three were significant in both healthy and OA MSCs (*ADAMTS4*, *MMP13* and *RUNX2* bn), four were significant only in healthy MSCs (*ACAN*, *SOX9*, *ADAMTS5* and *COL1A1*) and two were significant only in OA MSCs (*MMP1* and *RUNX2*) (Tables 5 and 8). The significant changes observed in gene expression however were not always in the same direction between the comparisons made.

Overall there are clear differences in the gene expression response of healthy and OA MSCs undergoing chondrogenesis to exogenous GDF5 but there are also several similarities. Conclusions derived from intra-group analysis are likely therefore to be more robust than those drawn from the inter-group analysis.

## Discussion

In this report we initially obtained MSCs from a total of seven healthy donors and 21 OA patients. The MSCs from these 28 individuals were firstly subjected to culture and expansion. For all seven healthy donors the cells expanded successfully whereas the success rate for the OA patients was just over half (11 of 21 patients). It has been reported that there is a high degree of donor-to-donor variability in the expansion capacity of MSCs [19] and our data would support this. We next undertook chondrogenic differentiation on those MSCs that had expanded. Three of the seven healthy donor MSCs and four of the remaining 11 OA MSCs successfully formed cartilage discs. Within the healthy donors and within the OA patients there were no clear differences with regard to age or sex between those who formed cartilage discs and those who did not. As far as we are aware, no other group has provided such explicit data on the attrition rate of MSCs using the Transwell chondrogenic system; 57% for our healthy donors (three of seven formed discs) and 81% for our OA donors (four of 21 formed discs). In the original manuscript that reported the Transwell protocol [15] the investigators analysed five young donors (aged 20–44 years and purchased from Lonza; comparable therefore to our healthy donors) and performed experiments on “cells from 2–5 donors”, which may imply attrition. Oda and colleagues [20] isolated MSCs from the knees (rather than the hips, as we have done here) of 29 OA patients and reported on the proliferation of seven, which again may imply attrition although this is an assumption as it is not clear whether the authors chose to focus only on these seven. Furthermore, these cells were not subsequently subjected to disc formation but were instead used for chondrogenic pellet analysis, and as such Transwell attrition cannot be inferred from the Oda report. Overall, seven of our original cohort of 28 healthy donors and OA patients generated cartilage discs, a combined attrition rate of 75%. We believe this to be the first such reported measure for the Transwell system using a reasonably large sample size. These seven discs were subsequently subjected to further study.

The cartilage discs from all seven stained positive for Safranin-O and Masson’s trichrome, demonstrating that the discs produce an extracellular matrix rich in proteoglycan and collagen. One of our key observations was that the cartilage discs have a greater wet mass when exposed to the variant A form of GDF5. This response was seen for healthy and OA MSCs. We used wet mass as a proxy for anabolism and our data therefore implies that chondrogenesis of MSCs is enhanced in the presence of this form of the growth factor. It should be noted that for OA patient E the discs showed a substantial increase in wet mass in response to GDF5 at day 3 but that by day 7 the wet mass had decreased, implying that GDF5 is not trophic in all contexts. We do not believe though that this invalidates our decision to use a one-tailed rather than a two-tailed t-test to analyse the disc wet mass data, as our hypothesis was that GDF5 would stimulate growth and that was the case except for patient E at day 7. Variant A has two point mutations in its sequence that substitute two methionine residues to valine residues at positions 453 and 456 [11,16]. It was designed to have increased specificity for the BMBR-IA receptor; in our study the gene for this receptor, *BMPR1A*, was expressed at a higher level during chondrogenesis relative to the other GDF5 receptor genes. Our data therefore suggests that selecting the appropriate form of GDF protein, guided by the expression levels of the receptor genes, can help elicit a greater anabolic response to the growth factor.

Wet mass measurements lack the granularity that comes with assessing changes in the expression levels of a panel of genes. Our analysis of anabolic, catabolic and hypertrophic genes revealed different responses within the healthy donor group and within the OA patient group, but also a number of consistent changes within each group. There are therefore concordant as well as discordant responses to GDF5. When we compared healthy donors to the OA patients, there were limited consistencies between the two groups. The healthy donor MSCs are derived from a different skeletal site than the OA patient MSCs (iliac crest versus hip) and they are younger (average of 30 years versus an average of 68 years). These differences may contribute to the differential responses observed between healthy and OA and could make intra-group analysis more reliable than inter-group analysis. Nevertheless, the fact that there were a number of consistent responses within each group does imply that the MSCs have a capacity to respond in a predictable manner during chondrogenesis in the Transwell system. The gene expression changes were not confined to being a pro-anabolic or an anti-catabolic response, with changes seen in the anabolic genes *ACAN* and *SOX9*, the catabolic genes *ADAMTS4*, *ADAMTS5*, *MMP1* and *MMP13*, and the hypertrophic genes *COL1A1* and *RUNX2*. Previous studies have shown exogenous GDF5 to have anabolic as well as hypertrophic effects in MSC pellet culture, with increased *ACAN*, *COL1A1* and *COL10A1* expression [12]. This finding may explain the consistent upregulation of the RUNX2 bone specific transcript *RUNX2* bn that we observed in both healthy and OA MSCs at day 3 in response to GDF5 variant; RUNX2 is a transcription factor that is essential for the regulation of chondrocyte proliferation, which occurs during the process of chondrocyte maturation and formation of endochondral bone [21].

At the individual donor or patient level, the greatest fold changes in expression (increases and decreases) at any gene were in response to GDF5 variant. In the healthy donors, exposure to this form of the growth factor also resulted in a larger, significantly increased expression of *SOX9* in cartilage discs compared to exposure to wildtype GDF5. These observations may be a reflection of the increased expression of *BMPR1A* making the MSCs particularly responsive to the variant A form of GDF5. It is also potentially noteworthy that the one donor/patient who demonstrated a decrease rather than an increase in disc wet mass between days 3 and 7 was the oldest person studied; donor E, aged 81 years and 12 years older than the second oldest patient, donor D. An analysis of a larger number of OA patients across the age range may clarify whether this observation is indicating a potential age effect.

In our study we undertook a range of analyses involving a number of different comparisons. The *p* values presented are not Bonferroni corrected. As noted above, one of our key observations was that the cartilage discs have a greater wet mass when exposed to GDF5 variant A, with a *p* of 0.0022 for day 3 and day 7 combined. If we multiply this *p* by the eight tests performed (three conditions (untreated, wildtype and variant A), two days (3 and 7) and three comparisons (days 3 and 7 combined, day 3 alone and day 7 alone)) it remains significant, with a value of 0.018 (0.0022 x 8). The lowest *p* value observed in our gene expression analysis was 0.0005 for the OA patients and *MMP13* following variant A treatment. This *p* would also remain significant following correction for the 18 tests performed in this analysis (two groups (healthy and OA), two conditions (wildtype and variant A) and 14 gene transcripts), with a value of 0.009 (0.0005 x 18). None of the other significant *p* values would survive Bonferroni correction although many would be close to significant, in the range of 0.1–0.05.

In summary, our results demonstrate that there is a consistent response to GDF5 in MSCs undergoing chondrogenesis, but that the responses differ between MSCs derived from healthy donors and OA patients. We demonstrated expression of all three GDF5 receptor genes, with the relative expression of each matching the results from our previous primary chondrocyte study [11], and with our analysis of wet mass as a gross measure of anabolic response highlighting the value of tailoring the GDF5 protein used to the receptors expressed. Unlike that earlier study of ours, in this current report we did not measure activation of SMAD signalling but instead inferred it in that GDF5 did act to promote growth. We have to acknowledge however that it is possible that SMAD signalling was not activated at all or that its activation may have differed between individuals, and that this could account for some of the inter-individual variability of the measures we have recorded. Equally, we did not measure expression of the GDF5 inhibitor noggin, which may fluctuate naturally between individuals, possibly in response to GDF5 treatment. Such potential differences in noggin levels could also have contributed to the inter-individual variability that we observed. With regards to tissue engineering and an attempt to alleviate the genetic susceptibility mediated by the rs143383 association signal. This signal’s effect is a reduction in the expression of the *GDF5* gene and as such one possible mechanism for overcoming this would be to provide cells with exogenous GDF5 protein. The fact that our data implies that OA MSCs will respond in a predictable, anabolic manner to such treatment offers support to investigate this possibility further.

Our study began with MSCs from a total of seven young donors and 21 OA patients but only seven of these (three donors and four patients) were subsequently able to generate cartilage discs, an attrition rate of 57% for donors, 81% for OA and 75% combined. These rates, which we believe derive from the largest such study yet undertaken for the Transwell system and which should therefore be considered as pilot or exploratory analyses, highlight the arduous nature of undertaking studies such as ours; for example, to create day 7 disc data from 50 OA patient MSCs would require an initial study group of 250. Nevertheless, we observed significant effects that survived Bonferroni correction and as such, our data supports further studies into the efficacy of the variant A form of GDF5 as a cartilage anabolic molecule.

## Conclusions

Unlike chondrocytes from OA patients, MSCs do respond in a predictable, anabolic manner during chondrogenesis to exogenous GDF5, and in particular to the variant A form of this growth factor. This may therefore provide a route to modulate the genetic deficit mediated by the rs143383 association signal that resides within the *GDF5* gene.

## Supporting information

S1 Fig Expression of the GDF5 receptor genes for each healthy donor and for each OA patient. This is the data of Fig 1 but presented for each of the individuals studied.
(DOCX)

S1 Table Primer and probe sequences of the TaqMan assays used for quantitative real time PCR. *RUNX2* bn, bone specific *RUNX2* transcript.
(DOCX)
