# Supplementary material for: A consistent and potentially exploitable response during chondrogenesis of mesenchymal stem cells from osteoarthritis patients to the protein encoded by the susceptibility gene GDF5
Source: PLoS One. 2017 May 8;12(5):e0176523. doi: 10.1371/journal.pone.0176523 (PMC5421763; doi:10.1371/journal.pone.0176523)
Supplement: S1 Fig — This is the data of Fig 1 but presented for each of the individuals studied. (DOCX) [file pone.0176523.s001.docx]

**S1 Fig. Expression of the GDF5 receptor genes for each healthy donor and for each OA patient.**

This is the data of Fig 1 but presented for each of the individuals studied. ****
