# Supplementary material for: A consistent and potentially exploitable response during chondrogenesis of mesenchymal stem cells from osteoarthritis patients to the protein encoded by the susceptibility gene GDF5
Source: PLoS One. 2017 May 8;12(5):e0176523. doi: 10.1371/journal.pone.0176523 (PMC5421763; doi:10.1371/journal.pone.0176523)
Supplement: S1 Table — RUNX2 bn, bone specific RUNX2 transcript. (DOCX) [file pone.0176523.s002.docx]

**S1 Table. Primer and probe sequences of the TaqMan assays used for quantitative real time PCR.**

*RUNX2* bn, bone specific *RUNX2* transcript.

| **Gene** | **Forward primer (5'-3')** | **Reverse primer (5'-3')** | **Probe (5'-3')** |
| --- | --- | --- | --- |
| *GAPDH* | ACATCGCTCAGACACCATG | TGTAGTTGAGGTCAATGAAGGG | AAGGTCGGAGTCAACGGATTTGGTC |
| *HPRT1* | TGCTGAGGATTTGGAAAGGG | ACAGAGGGCTACAATGTGATG | AGGACTGAACGTCTTGCTCGAGATG |
| *18s* | CGAATGGCTCATTAAATCAGTTATGG | TATTAGCTCTAGAATTACCACAGTTATCC | TCCTTTGGTCGCTCGCTCCTCTCCC |
| *BMPR2* | GGCTGACTGGAAATAGACTGG | CACAGTCCCTCAAGTTCACAG | CCTCGCTTATGGCTGCATTATCTTCCTC |
| *BMPR1A* | ACAAAGTTCTGGTAGTGGGTC | CATCCATACTTCTCCATATCGGC | ATTCAGATGGTCCGGCAAGTTGGT |
| *BMPR1B* | CTGCACAGAAAGGAACGAATG | AGGACCAAGAGCAAACTACAG | TGGAGGCAGTGTAGGGTGTAGGT |
| *ACAN* | TGTGGGACTGAAGTTCTTGG | AGCGAGTTGTCATGGTCTG | CTGGGTTTTCGTGACTCTGAGGGT |
| *COL2A1* | ACCTTCATGGCGTCCAAG | AACCAGATTGAGAGCATCCG | AGACCTGAAACTCTGCCACCCTG |
| *SOX9* | CTGGTACTTGTAATCCGGGTG | ACTTGCACAACGCCGAG | TCTGGAGACTTCTGAACGAGAGCGA |
| *TIMP1* | TTCTGCAATTCCGACCTCG | TCATAACGCTGGTATAAGGTGG | TTGACTTCTGGTGTCCCCACGAAC |
| *ADAMTS4* | CTGGGTATGGCTGATGTGG | TGGCTTGGAGTTGTCATGG | TTCACTGCTGCTCATGAACTGGGT |
| *ADAMTS5* | CAAGTGCGGAGTATGTGGAG | GTCTTTGGCTTTGAACTGTCG | TTTATGTGGGTTGCCCCTTCAGGA |
| *MMP1* | AAGATGAAAGGTGGACCAACAATT | CCAAGAGAATGGCCGAGTTC | CAGAGAGTACAACTTACATCGTGTTGCGGCTC |
| *MMP13* | AAATTATGGAGGAGATGCCCATT | TCCTTGGAGTGGTCAAGACCTAA | CTACAACTTGTTTCTTGTTGCTGCGCATGA |
| *BGLAP* | CAGCGAGGTAGTGAAGAGAC | TGAAAGCCGATGTGGTCAG | TCCCAGCCATTGATACAGGTAGCG |
| *COL1A1* | CCCCTGGAAAGAATGGAGATG | TCCAAACCACTGAAACCTCTG | TTCCGGGCA ATCCTCGAGCA |
| *COL10A1* | CAAGGCACCATCTCCAGG | TGGGCATTTGGTATCGTTCAG | ACTCCCAGCACGCAGAATCCAT |
| *IHH* | ATGAAGGCAAGATCGCTCG | GATAGCCAGCGAGTTCAGG | TCAAGGACGAGGAGAACACAGGC |
| *RUNX2* | AGCAAGGTTCAACGATCTGAG | TGAAGACGGTTATGGTCAAGG | CGGAGTGGACGAGGCAAGAGTTTC |
| *RUNX2* bn | AATGGTTAATCTCCGCAGGTC | TGTTTGATGCCATAGTCCCTC | CTGTTGGTCTCGGTGGCTGGTAG |
